# Mild behavioral impairment: A prodromal stage of dementia

**DOI:** 10.1590/S1980-57642009DN20400004

**Published:** 2008

**Authors:** Fernando E. Taragano, Ricardo F. Allegri, Constantine Lyketsos

**Affiliations:** 1 Servicio de Neuropsicología (SIREN), y Unidad de Investigación “René Barón” del Instituto Universitario CEMIC, Buenos Aires, Argentina.; 2 Division of Geriatric Psychiatry and Neuropsychiatry, Johns Hopkins University and Hospital, Baltimore, USA.

**Keywords:** mild behavioral impairment, dementia, Alzheimer’s disease, conversion, frontotemporal dementia

## Abstract

Mild cognitive impairment (MCI) was defined by Petersen et al. (1999) as progressive memory loss, a prodrome of Alzheimer’s disease. MCI is a well-established entity that can be both a diagnosis in medical practice and a valid target of Alzheimer’s prevention therapy. More recently MCI has expanded to include other cognitive domains with other potential causes: amnestic MCI, multiple domains MCI, and single domain non-amnestic MCI. Behavioral symptoms in MCI are associated with a higher risk of dementia, but their association with dementia risk in patients without MCI is unknown. The objective of our paper was to address the question of whether aging patients with behavioral symptoms with or without cognitive impairment represent a population at risk for dementia. Mild Behavioral Impairment (MBI) defines a late life syndrome with prominent psychiatric and related behavioral symptoms in the absence of major cognitive symptoms. MBI also appears to be a transitional state between normal aging and dementia. MBI may carry a higher risk for dementia than MCI. A subgroup of MBI patients is likely to exhibit symptoms of a frontotemporal dementia (FTD) prodrome. We proposed 4 subtypes of patients at risk for dementia: amnestic MCI (which is said to progress preferentially to Alzheimer’s disease), multiple domain MCI (which may represent normal aging or may progress to vascular cognitive impairment or a neurodegenerative disorder), single domain non-amnestic MCI, and MBI (which may progress to frontotemporal dementia, Lewy Body dementia or Alzheimer’s disease). We concluded that MBI is a counterpart of MCI as a transitional state between normal aging and dementia. These findings have implications for early detection, prevention, and treatment of patients with late-life dementia.

## Abstract (pt)

Comprometimento cognitivo leve (CCL) foi definido por Petersen et al. (1999) como uma perda progressiva da memória, pródromo da doença de Alzheimer. CCL é uma entidade bem estabelecida que tanto pode ser um diagnóstico na prática clínica como um alvo válido para terapias preventivas da doença de Alzheimer. Recentemente, o CCL expandiu-se para incorporar outros domínios cognitivos com outras causas potenciais: CCL amnésico, de múltiplos domínios e de um único domínio não-amnéstico. Sintomas comportamentais no CCL são associados com risco mais elevado de demência, mas sua associação com o risco de demência na ausência de comprometimento cognitivo não é conhecida. O objetivo deste artigo foi o de verificar se pacientes idosos com sintomas comportamentais constituem população de risco para demência. Comprometimento comportamental leve caracteriza-se como uma síndrome que se manifesta em idosos constituída por sintomas psiquiátricos e sintomas comportamentais relacionados na ausência de sintomas cognitivos mais evidentes. O comprometimento comportamental leve parece ser um estado de transição entre o envelhecimento normal e demência e pode conferir um risco maior para demência do que o CCL. Um subgrupo de pacientes com comprometimento comportamental leve provavelmente está na fase prodrômica de demência frontotemporal (DFT). Nós propomos que se considerem quatro grupos de pacientes com risco de demência: CCL amnéstico (que segundo se admite evolui preferencialmente para doença de Alzheimer), CCL de múltiplos domínios (que pode representar envelhecimento normal ou pode evoluir para comprometimento cognitivo vascular ou para doença neurodegenerativa), CCL de único domínio não-amnéstico e Comprometimento Comportamental Leve (que pode evoluir para DFT, demência com corpúsculos de Lewy ou doença de Alzheimer). Concluímos que o Comprometimento Comportamental Leve é uma complementação ao CCL como um estado de transição entre o envelhecimento normal e demência. Estes achados têm implicações para a detecção precoce, prevenção e tratamento de demência de instalação tardia.

Alzheimer’s disease (AD) is a globally widespread chronic condition that seriously affects patients, their families and society.^1,2^ An understanding of prodromal stages or early clinical presentations of AD is a significant priority since it would aid early detection, facilitate early treatment, and lead to prevention. There is a clinical cognitive continuum from normal aging to AD. Cognitive decline without dementia has been commonly considered to be a normal consequence of brain aging, but it can also indicate the onset of dementia. The boundary between normal aging and very early AD is becoming a major focus of research. Pre-dementia diagnosis is closely connected with the development of AD prevention therapies.

Many attempts have been made to define aging-related cognitive decline. The idea of aging-effects versus disease is not new; in 1962, Kral et al.,^3^ described “benign senescent forgetfulness” (BSF) in which fairly unimportant details of an experience (e.g. a name, a place or a date) are not recalled but do not interfere with activities of daily living and do not progress to dementia. Kral also recognized that “differentiation of the benign and malignant types of senescent forgetfulness does not necessarily mean that there are two neuropathological processes”.^3^ These diagnostic criteria were imprecise, and had not been validated in controlled longitudinal studies. These cognitive changes in aging have been assigned various terms, such as age-associated memory impairment,^4^ late-life forgetfulness^5^ and aging-associated cognitive decline.^6^ These terms have been used largely to explain the extremes of normal aging, to characterize individuals who are neither normal nor demented. Such terms were criticized for being inaccurate.

Mild cognitive impairment was initially described in the late 1990s by Flicker and colleagues^7^ and denoted persons met criteria for stage 3 on the Global Deterioration Scale (GDS)^8^ or 0.5 on the Clinical Dementia Rating (CDR) instrument.^9^ GDS and CDR are severity rating scales and not diagnostic instruments. Both stages may correspond to MCI or may describe individuals with very mild dementia.^10^ Flicker proposed a clinical continuum from normal aging through mild cognitive impairment to Alzheimer’s disease. MCI was not normal aging: this construct was intended to be a clinical description of persons who were expected to develop AD.^11^

The identification of persons at risk of developing dementia, particularly Alzheimer’s disease, is of major economic importance, especially if preventive strategies or therapeutic actions are to be developed. This challenge explains the popularity of the concept of MCI and its wide application in the epidemiological, clinical, para-clinical and therapeutic domains.

Petersen diagnostic criteria for Mild Cognitive Impairment^11^ included: memory complaint preferably corroborated by an informant, memory impairment relative to age and education-matched healthy individuals, preserved general cognitive function, intact activities of daily living and no clinical evidence of dementia.

It is clear that the chosen definition of cognitive impairment will have a major impact on prevalence estimates. This impact could also extend to prognosis although these definitions describe syndromes and do not address causality.^12^

The international working group on MCI criteria^13^ included: the individual is neither normal nor demented; evidence of cognitive impairment, as shown by objectively measured decline over time or subjective report of decline by self or informant in conjunction with objective cognitive deficits; preserved activities of daily living and intact or minimally impaired complex instrumental functions. These criteria expand the construct of MCI to cognitive domains beyond memory and present MCI as a prodrome of multiple types of dementia.^14^

De Kosky and Chertkow^15^ proposed 3 subtypes of MCI: amnestic MCI, multiple domains MCI, and single domain non-amnestic MCI. Supporting studies are underway to determine whether amnestic and non-amnestic MCI have different prognoses of progression to dementia and which type of dementia they predict.^16^ They propose that amnestic MCI progresses preferentially to Alzheimer’s disease, multiple domains MCI may represent normal aging or may progress to vascular cognitive impairment or a neurodegenerative disorder, while single domain non-amnestic MCI may progress to frontotemporal dementia, Lewy Body dementia or Alzheimer’s disease (Figure 1).

**Figure 1:**
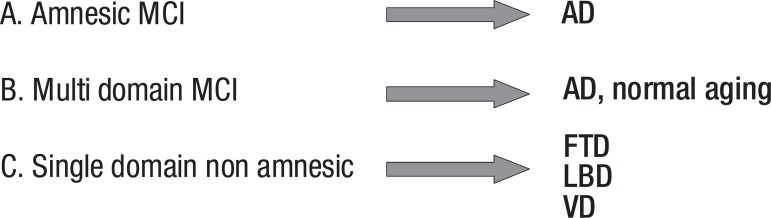
Subtypes of mild cognitive impairment. MCI, mild cognitive impairment; AD, Alzheimer’s disease; FTD, frontotemporal dementia; LBD, Lewy Body disease; VD, vascular dementia.

In clinical-based studies, the typical rate at which MCI patients progress to AD is 10 to 15% per year in contrast with the incidence rates for the development of dementia in normal elderly subjects of 1–2% per year.^11^ In population-based studies, the prognosis of MCI deficits seems much less ominous. Ritchie et al.^17^ found that only 22% of MCI subjects developed degenerative dementia over an 8-year follow-up period. Rates of progression are widely discrepant across studies and populations.^17-20^ These discrepancies are partly related to the nature of the populations (clinical/referral vs. community-based) and length of follow-up. They also seem to be due, in large part, to the different definitions and inclusion criteria that used in the studies^18^ Most amnesic MCI patients who died appeared to have been transitional between the neuropathological changes found in aging and characteristics of very early AD.^21^

We could hypothesize that, at least for some patients, the natural clinical course of Alzheimer could be “presymptomatic AD”, “pre-dementia AD or MCI of AD type” and “Dementia of AD” (Figure 2).

**Figure 2:**
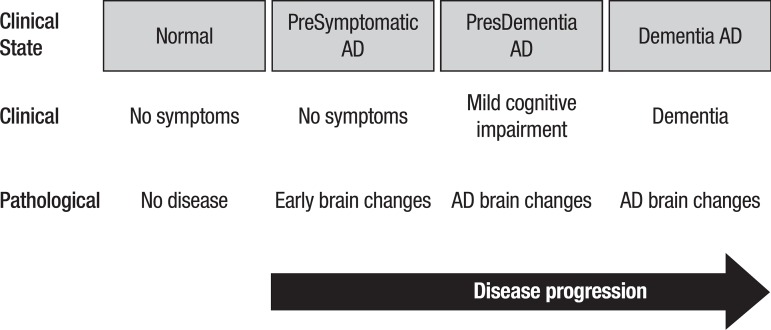
Hypothesized natural course of Alzheimer’s disease.

In the last several years, there has been growing awareness of the importance of neuropsychiatric symptoms (NPchS) in dementia, given their near universal occurrence over the course of dementia, associated caregiver burden, and correlation with early institutionalization. Whereas dementia is still defined as a cognitive disorder, neuropsychiatric symptoms are now regarded as an intrinsic aspect of dementia, and the underlying causes usually as neurodegenerative processes.

Although neuropsychiatric symptoms are common in dementia^22-26^ they have received less attention in the prodromal stages of dementia. In a population-based study, the most common neuropsychiatric symptoms in MCI were apathy, depression, agitation, delusions, hallucinations, and sleep impairment.^27,28^ In MCI patients, the occurrence of neuropsychiatric symptoms is associated with a higher risk for the onset of dementia. For example, depression in MCI has been reported to double the risk of dementia.^28,29^ Furthermore, cognitively normal elderly individuals who develop depression are at increased risk for subsequent MCI.^30^ However, not all prodromal stages involve prominent cognitive impairment. Many patients develop neuropsychiatric symptoms as the first indicator of impending dementia. This is most common in patients with FTD, but it is also the case in patients with AD. For example, we reported that psychiatric symptoms were the first indication of change, before the occurrence of cognitive symptoms, in 50% of all dementia patients who consulted our service. Of these patients, 36% had FTD, 28% had AD; 18% had VaD and 18% had other types of dementia.^31^ As a result, we proposed the “Mild Behavioural Impairment” (MBI) syndrome, consisting of: persistent behavioral changes and mild psychiatric symptoms, especially disinhibition; non serious cognitive complaints; normal activities of daily living; and absence of dementia.^31-35^.

Between January 2001 and January 2006, a new consecutive series of 1491 elderly outpatients were evaluated in our Unit (CEMIC Cohort). After a thorough neuropsychiatric assessment, 425 were found to present with cognitive and/or behavioral symptoms; 119 of these were found to have MBI and 239, MCI, whereas 17 presented with late onset psychosis. The median follow up was 30 months.^34,35^.

Graph 1 compares age-adjusted Kaplan-Meier plots of the time to conversion to dementia between both groups. Conversion was faster in MBI patients.

**Graph 1:**
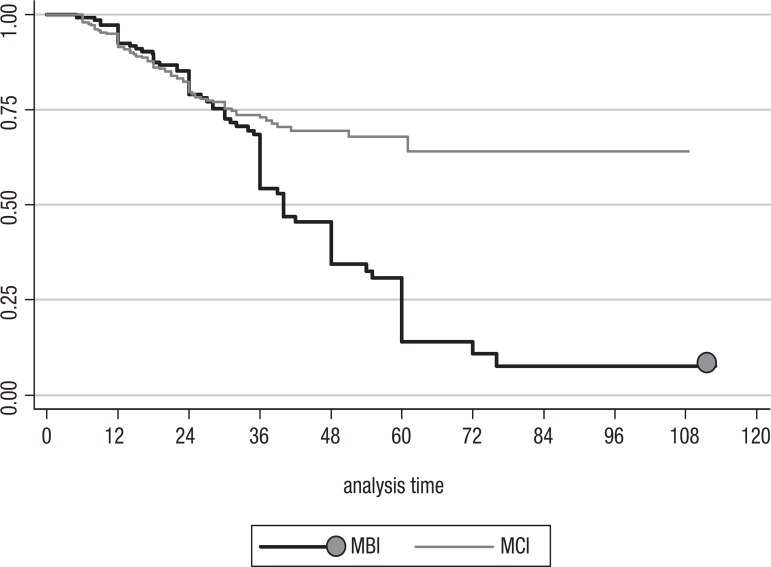
Age-adjusted survival functions by initial diagnosis. Kaplan Meier Survival Analysis. Analysis time: in months. MCI, mild cognitive impairment; MBI, mild behavioral impairment.

The MBI group converted to dementia much faster than the MCI group. MBI patients were more likely to convert to FTD (44.5%), AD (22.7%) and LBD (4.2%).

An analysis was performed to compare rates of conversion to dementia in the following groups:

a) MCI without neuropsychiatric symptoms,
b) MCI with neuropsychiatric symptoms,
c) MBI with cognitive symptoms
d) MBI without cognitive symptoms.

MCI without NPS, and MBI without cognitive symptoms were quite different in terms of time to dementia onset, which proved much faster for MBI without cognitive symptoms than for MCI without NPS (log-rank test Χ^2^(3)=42.87 p<0.001) and MCI patients with psychiatric symptoms differed from MCI patients with no psychiatric symptoms in that they had a 4- fold greater risk of conversion to dementia (HR 4.01, 95% Conf. Interval 2.5-6.3) (Graph 2)

**Graph 2:**
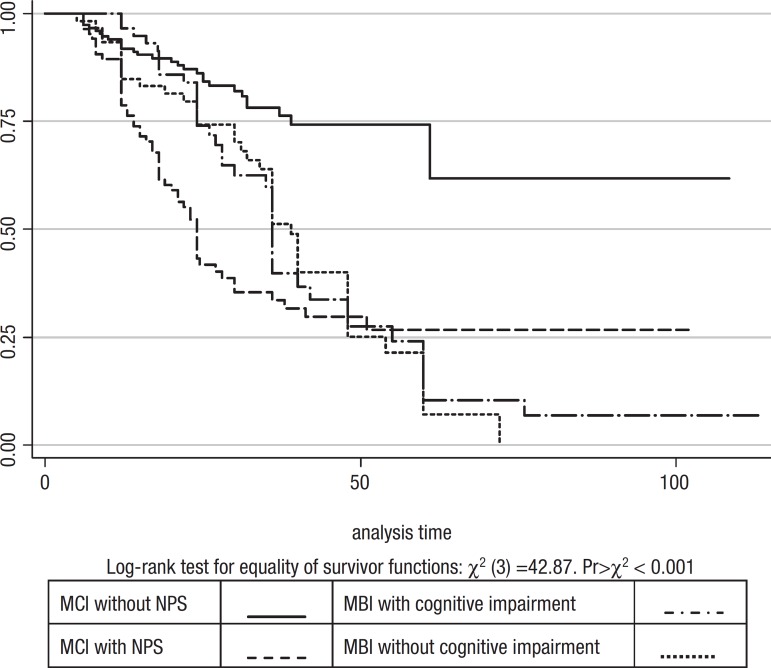
Kaplan-Meier estimate suvival by diagnosis.

MBI patients developed frontotemporal dementia more often than other types of dementia. Overall, in both MCI and MBI groups, the presence of neuropsychiatric symptoms (with or without cognitive symptoms) was more strongly associated with conversion to dementia than the presence or severity of cognitive impairment. Patients with MBI experienced a more rapid conversion to dementia than those with MCI.

We proposed 4 subtypes of patients at risk for dementia: amnestic MCI (which is said to progress preferentially to Alzheimer’s disease), multiple domain MCI (which may represent normal aging or may progress to vascular cognitive impairment or neurodegenerative disorder), single domain non-amnestic MCI (which may progress to fronto-temporal dementia, Lewy Body dementia or Alzheimer’s disease) and MBI (which may progress preferentially to fronto-temporal dementia, Lewy Body dementia, or Alzheimer’s disease (Figure 3).

**Figure 3:**
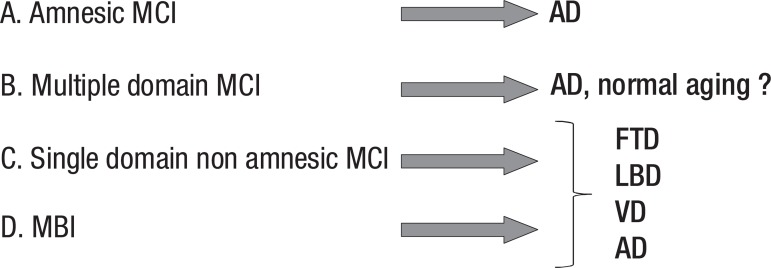
Subtypes of patients at risk for dementia. MCI, mild cognitive impairment; AD, Alzheimer’s disease; FTD, frontotemporal dementia; LBD, Lewy Body disease; VD, vascular dementia.

## Conclusions

We concluded that MBI is a counterpart of MCI as a transitional state between normal aging and dementia. MBI carries a higher risk for conversion to dementia than MCI. This review emphasizes the importance of neuropsychiatry symptoms as critical aspects of late-life cognitive disorders. A better understanding of these observations may contribute to early detection of dementia and therapy targets, and possibly enable prevention..
